# Effects of Digital Device Ownership on Cognitive Decline in a Middle-Aged and Elderly Population: Longitudinal Observational Study

**DOI:** 10.2196/14210

**Published:** 2019-07-29

**Authors:** Yinzi Jin, Mingxia Jing, Xiaochen Ma

**Affiliations:** 1 Department of Global Health School of Public Health Peking University Beijing China; 2 Department of Public Health Shihezi University School of Medicine Xinjiang China; 3 China Center for Health Development Studies Peking University Beijing China

**Keywords:** digital access, cognitive decline, China

## Abstract

**Background:**

Cognitive decline is a major risk factor for disability and death and may serve as a precursor of dementia. Digital devices can provide a platform of cognitively stimulating activities which might help to slow cognitive decline during the process of normal aging.

**Objective:**

This longitudinal study aimed to examine the independent protective factors of desktop and cellphone ownership against cognitive decline in mid-life and older adulthood and to examine the combined effect of desktop and cellphone ownership on the same outcome.

**Methods:**

Data was obtained from a China Health and Retirement Longitudinal Studies (CHARLS) cohort made up of 13,457 community-dwelling adults aged 45 years or above in 2011-2012. They were followed for 4 years, with baseline measurements taken as well as 2 two-year follow-up visits. Cognitive function was tested during the baseline test and follow-up visits. A global cognition z-score was calculated based on two domains: word recall and mental intactness. The key independent variables were defined as: whether one had desktops with internet connection at home and whether one had a cellphone. An additional categorical variable of three values was constructed as: 0 (no desktop or cellphone), 1 (desktop or cellphone alone), and 2 (desktop and cellphone both). Mixed-effects regression was adjusted for demographic and health behavior as well as health condition risk factors.

**Results:**

Adjusted for demographic and health behavior as well as health condition risk factors, desktop and cellphone ownership were independently associated with subsequent decreased cognitive decline over the four-year period. Participants without a desktop at home had an adjusted cognitive decline of –0.16 standard deviations (95% CI –0.18 to –0.15), while participants with a desktop at home had an adjusted cognitive decline of –0.10 standard deviations (95% CI –0.14 to –0.07; difference of –0.06 standard deviations; *P*=.003). A similar pattern of significantly protective association of 0.06 standard deviations (95% CI 0.03-0.10; *P*<.001) between cellphone ownership and cognitive function was observed over the four-year period. Additionally, a larger longitudinal protective association on cognitive decline was observed among those with both of the digital devices, although the 95% CIs for the coefficients overlapped with those with a single digital device alone.

**Conclusions:**

Findings from this study underscored the importance of digital devices as platforms for cognitively stimulating activities to delay cognitive decline. Future studies focusing on use of digital devices are warranted to investigate their longitudinal protective factors against cognitive decline at mid- and later life.

## Introduction

Cognitive decline is a major risk factor for physical disability [1] and death [2] and may serve as a precursor of dementia [3,4]. Cognitive decline, which often begins in individuals aged between 45-60 years, is an irreversible pathophysiological process of brain change [5]. In China, the burden of cognitive decline is increasing as a result of populations aging rapidly. Recent studies show that almost 20% of Chinese adults aged 60 or above have mild cognitive impairment, of which 6% progress to dementia annually [6-8]. However, resources for the care of those elderly with cognitive decline are insufficient, raising public health concerns about suboptimal geriatric medicine and psychiatric care services [9].

To address this challenge of cognitive decline, innovative strategies for interventions are needed. A growing number of epidemiologic studies have clearly suggested that digital devices, like internet-based instruments, can provide a platform of cognitively stimulating activities which might help to slow the path of cognitive decline in the process of normal aging [10-12]. Longitudinal studies and meta-analyses in developed countries have shown that the use of the internet can be a protective factor for cognitive decline among the older population [13-16]. In the context of China, there has been a marked increase in internet access among mid-aged and elderly populations [17], although the national data on internet use is limited. Given the potential protective association between internet use and cognitive decline, two questions remain unanswered in China. First, does the protective association between digital devices and cognitive decline found in other settings apply to mid-aged and elderly populations in China, where access to digital devices and the aging population are both growing rapidly? Second, given the diverse digital instruments that provide internet connection through which different activities might be facilitated, what is the combined effect of multiple digital instruments such as desktops and cellphones?

This study used a nationally representative longitudinal survey to: (1) investigate whether desktop and cellphone ownership might be independently associated with decreased cognitive decline in mid-life and older adulthood; and (2) to examine the combined effect of both of the digital devices on cognitive decline.

## Methods

### Design, Setting, and Participants

Participants were enrolled in the China Health and Retirement Longitudinal Study (CHARLS), a biennial prospective observational study, nationally representative of Chinese adults aged ≥45 years and their spouses. This study includes assessments of social, economic, and health circumstances of community residents from 2011-2012 [18]. Data from four years of follow-up visits (2011-2012, 2013-2014, 2015-2016) were utilized. Of the 13,548 participants who underwent cognitive assessment in the baseline wave (2011-2012), 91 participants were excluded due to lack of data on desktop and cellphone information. Among 13,457 participants enrolled as our analytic (baseline) cohort, a lot of 2073 (15.4%) were lost to follow-up due to not attending one or more follow-up surveys during the four years of the study period. Potential reasons for attrition included death or dropping out from the study. All participants provided written informed consent, and survey protocols were approved by the Peking University Ethics Review Board [19].

### Measurements

#### Cognitive Function

Cognitive functions were tested during the baseline test and during the two follow-up in-person household visits in a two-year interval. Following a previous study on CHARLS [20], the present study used a global cognitive function score based on two domains of cognitive functions: word recall and mental intactness. For word recall, each participant was asked to repeat as many of the 10 Chinese nouns just read to them as possible (immediate word recall) and then to recall the same 10-word list 5 minutes later (delayed recall). Answers to these questions were aggregated into a single word recall score ranging from 0 to 10. For the mental intactness domain, measures including numerical ability, time orientation, and picture drawing were used to formulate the score. Numerical ability was measured by serial 7 subtractions from 100 (up to five times), time orientation was measured by naming that day’s date (month, day, year and date of week), and picture drawing was measured by asking each participant to redraw a picture of two overlapping pentagons shown to them. Answers to these questions were then aggregated into a single mental intactness score ranging from 0 to 10. To aid in comparison of cognitive test results, z-scores (the difference between participant’s score and the sample mean, divided by the standard deviation of the baseline sample) were created for both domain-specific cognitive function of word recall and mental intactness. A global z-score was calculated according to the means and standard deviations of two domain-specific z-scores. The details of implementation procedures (interviewer recruitment, training and material preparation, quality control and data cleaning) were added in Multimedia Appendix 1.

#### Internet Access

The key independent variables were defined as whether there was the presence of desktops with internet connection at home (desktop ownership) and whether an individual had a cellphone (cellphone ownership). Because the survey did not ask whether the cellphone was a smartphone or a mobile phone without internet connection, this study’s definition of cellphone ownership refers to mobile phones both with and without internet connection. Participants with both desktop and cellphone ownership, as defined above, were classified as having combined digital device ownership. Because only 1.23% (166/13,457) of our sample had desktops alone, a categorical variable of three values was then constructed as 0 (no desktop or cellphone), 1 (desktop or cellphone alone), and 2 (desktop and cellphone both).

#### Covariates

Following previous studies, there were several covariates that were included in the present study [21-23]. They include demographic and socioeconomic status covariates such as age, gender (male or female), educational attainment (illiterate, part of primary school, primary school, middle school, high school or above), marital status (married, widowed, separated, divorced, or never married), and residence status (currently living in rural or urban area). In addition, there were health behavior covariates which included smoking status (current, former or never) and alcohol drinker (current, former or never). Lastly, there were health condition covariates included, such as whether or not the participant self-reported whether they had ever been diagnosed with the following diseases: high blood pressure, diabetes, or stroke.

### Statistical Analysis

A descriptive analysis presented the characteristics of study participants among the full sample as a whole and then by subsamples of desktop and cellphone ownership status. Two pairs of linear mixed-effect models were used to assess the longitudinal association of digital access and cognitive function over time. The first pair of models focused on desktop and cellphone ownership as independent predictors, and the second pair of models focused on the combined effect of having both desktop and cellphone or the single effect of having the desktop or cellphone alone, compared to having no desktop or cellphone. Stratified analysis by age and gender was conducted to test differential associations across subgroups. All models were adjusted for demographic (age, sex, education, marriage, rural or urban residence) and health behavior (smoke, drink) as well as health condition risk factors (self-reported hypertension, diabetes, and stroke). In order to evaluate the potential bias due to non-response and attrition across difference survey waves, sensitivity analysis was conducted using the same models with the complete sample who participated in all three waves. All analyses were conducted in Stata 14.1 (StataCorp LP).

## Results

### Characteristics of Participants

Data from 13,457 participants aged 45 or above (mean age 58.7, SD 9.37) were included for analysis. At baseline, participants with a desktop were more likely to be younger, female, better educated, either married or partnered, and less likely to be living in a rural area and smoke than participants without desktop ownership. Fewer differences were observed between participants with and without cellphones, however participants with cellphones were more likely to be younger, better educated, and either married or partnered Table 1. We also compared the baseline characteristics across three groups (without desktop or cellphone, with desktop or cellphone only, and with both desktop and cellphone) in Multimedia Appendix 2.

**Table 1:** Characteristics of the study participants.

Characteristic		Total (N=13,457)	Desktop Ownership			Cellphone Ownership		
			No (n=11,143)	Yes (n=2314)	*P* value	No (n=2764)	Yes (n=10,693)	*P* value
Age, mean (SD)		58.7 (9.37)	59.3 (9.43)	55.6 (8.44)	<.001	64.8 (9.42)	57.1 (8.68)	<.001
Male sex, n (%)		6590 (49.0)	5508 (49.4)	1082 (46.8)	.02	1339 (48.4)	5251 (49.1)	.53
**Educational level, n (%)**					**<.001**			**<.001**
	Illiterate	3340 (24.8)	3106 (27.9)	234 (10.1)		1133 (41.0)	2207 (20.6)	
	Part of primary school	2462 (18.3)	2208 (19.8)	254 (11.0)		562 (20.3)	1900 (17.8)	
	Primary school	2937 (21.8)	2549 (22.9)	388 (16.8)		543 (19.7)	2394 (22.4)	
	Middle school	2924 (21.7)	2251 (20.2)	673 (29.1)		344 (12.5)	2580 (24.1)	
	High school or above	1793 (13.3)	1029 (9.2)	764 (33.0)		181 (6.6)	1612 (15.1)	
**Marital status, n (%)**					**<.001**			**<.001**
	Married or Partnered	11,863 (88.2)	9717 (87.2)	2146 (92.7)		2227 (80.6)	9636 (90.1)	
	Otherwise	1594 (11.8)	1426 (12.8)	168 (7.3)		537 (19.4)	1057 (9.9)	
Rural residence, mean (SD)		0.59 (0.49)	0.66 (0.47)	0.26 (0.44)	<.001	0.66 (0.48)	0.57 (0.49)	<.001
**Smoke, n (%)**					**<.001**			**.69**
	Current	4205 (31.3)	3578 (32.1)	627 (27.1)		844 (30.5)	3361 (31.4)	
	Former	1237 (9.2)	1035 (9.3)	202 (8.7)		276 (10.0)	961 (9.0)	
	Never	8013 (59.6)	6528 (58.6)	1485 (64.2)		1643 (59.5)	6370 (59.6)	
**Alcohol drinker, n (%)**					**<.001**			**.01**
	Current	4492 (33.4)	3684 (33.1)	808 (34.9)		856 (31.0)	3636 (34.0)	
	Former	1140 (8.5)	987 (8.9)	153 (6.6)		262 (9.5)	878 (8.2)	
	Never	7821 (58.1)	6468 (58.1)	1353 (58.5)		1646 (59.6)	6175 (57.8)	
Ever had high blood pressure, mean (SD)		0.3 (0.44)	0.3 (0.44)	0.3 (0.44)	.87	0.3 (0.46)	0.3 (0.44)	<.001
Ever had diabetes, mean (SD)		0.1 (0.25)	0.1 (0.24)	0.1 (0.27)	.01	0.1 (0.25)	0.1 (0.25)	.33
Ever had stroke, mean (SD)		0.0 (0.16)	0.0 (0.16)	0.0 (0.15)	.26	0.0 (0.17)	0.0 (0.15)	.02

### Independent Association Between Desktop or Cellphone Ownership and Cognitive Function Over Time

**Figure 1:**
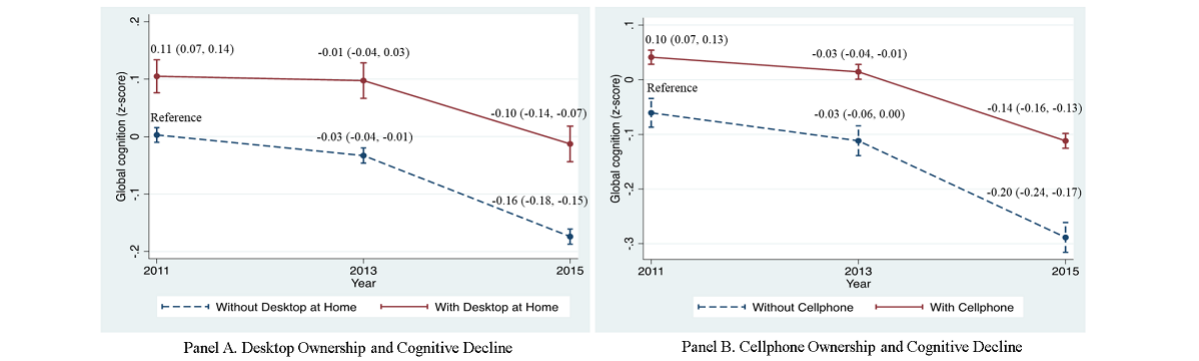
Independent association between desktop or cellphone ownership and cognitive function over time. Adjusted for demographic (age, sex, education, marriage, rural or urban residence) and health behavior (smoke, drink) as well as health condition risk factors (self-reported hypertension, diabetes, and stroke).

In mixed-effects regression adjusted for demographic and health behavior as well as health condition risk factors, desktop or cellphone ownership were associated with similarly higher baseline global cognitive scores. Compared with participants without a desktop at home, participants with desktop ownership had global cognitive scores at baseline that were 0.11 standard deviations (95% CI 0.07-0.14) higher. Similar positive association was found between cellphone ownership and baseline global cognitive scores of 0.10 (95% CI 0.07-0.13) (Figure 1).

We then investigated whether desktop or cellphone ownership was independently associated with subsequent cognitive trajectories, adjusted for demographic and health behavior as well as health condition risk factors. Compared to participants without desktop ownership, there was a significant reduction in cognitive decline among participants with desktop ownership in the four-year follow-up. On average, over the two-year follow-up, participants without a desktop at home had an adjusted cognitive decline of –0.03 standard deviations (95% CI –0.04 to –0.01), while participants with a desktop at home had an adjusted cognitive decline of –0.01 standard deviations (95% CI –0.04 to 0.03). However, this difference of –0.02 standard deviations was not significant (*P*=.12). Over the four-year follow-up, participants without a desktop at home had an adjusted cognitive decline of –0.16 standard deviations (95% CI –0.18 to –0.15), while participants with a desktop at home had an adjusted cognitive decline of –0.10 standard deviations (95% CI –0.14 to –0.07; difference of –0.06 standard deviations; *P*=.003) (Figure 1).

A similar pattern of significantly protective association of 0.06 standard deviations (95% CI 0.03-0.10; *P*<.001) between cellphone ownership and cognitive function was found over the four-year period while no significant difference was observed in the two-year follow-up period (Figure 1).

### Combined Effect of Desktop and Cellphone Ownership on Cognitive Function Over Time

In the next subsection we investigated the combined effect of desktop and cellphone ownership, adjusted for demographic and health behavior as well as health condition risk factors. In the baseline measured global cognitive score, participants having desktop or cellphone alone were 0.10 standard deviations (95% CI 0.07-0.13) higher and participants having both desktop and cellphone were 0.20 standard deviations (95% CI 0.15-0.24) higher than participants having neither desktop nor cellphone (Table 2).

Similar to the patterns in the independent association between desktop or cellphone ownership and cognitive decline, the longitudinal protective association was found in the longer-term four-year follow-up. Compared to participants having neither desktop nor cellphone, greater longitudinal protective association was observed among participants having both desktop and cellphone (SD 0.10, 95% CI 0.05-0.15). Although the effect size was larger than those participants having desktop or cellphone alone (SD 0.07, 95% CI 0.03-0.10), the two 95% CIs overlapped with each other (Table 2).

**Table 2:** Adjusted longitudinal association between digital device ownership and cognitive function over time (Total Number of Observations=33,956).

Variables		Point Estimates (95% CI)	*P* value
**Baseline Cognitive Function**			
	No Desktop or Cellphone	reference	
	Desktop or Cellphone Alone	0.10 (0.07-0.13)	<.001
	Desktop and Cellphone Both	0.20 (0.15-0.24)	<.001
**Longitudinal Protective Association in 2 Years**			
	No Desktop or Cellphone	reference	
	Desktop or Cellphone Alone	0.02 (–0.02 to 0.05)	.39
	Desktop and Cellphone Both	0.02 (–0.02 to 0.08)	.30
**Longitudinal Protective Association in 4 Years**			
	No Desktop or Cellphone	reference	
	Desktop or Cellphone Alone	0.07 (0.03-0.10)	<.001
	Desktop and Cellphone Both	0.10 (0.05-0.15)	<.001

### Stratified Analysis and Sensitivity Analysis

Our stratified analysis found that the protective association between digital device ownership and cognitive decline during the four-year follow-up was primarily driven by females (Multimedia Appendix 3) and relatively younger adult participants aged 45-59 (Multimedia Appendix 4). Sensitivity analyses restricted analytic cohorts to those 11,384 who participated in all survey waves. We found similar results with the original analysis (Multimedia Appendix 5).

## Discussion

### Principal Findings

This study is, to our knowledge, the first nationally representative study to show the longitudinal protective association between digital device ownership and cognitive function among mid-aged and elderly populations in China. China has the largest population aged 65 years and older in the world, and in 2050 the proportion of older persons within the total national population is projected to be around 25% [24]. The health needs for elderly people with irreversible cognitive impairment challenge China's health and social care system in a serious and unprecedented way. Over the past decade the proportion of the population connected to the internet has been growing exponentially, and digital device ownership shows potential for older people because it enables easier access to experiential, intelligential, social or emotional support for health purposes [25]. Taking advantage of the population-based nationally representative survey, we were able to investigate whether the preventive effect of desktop or cellphone ownership on cognitive function found in other settings still holds in the context of China, where access to internet and population aging are simultaneously increasing, and whether different digital devices have a combined effect on cognitive decline. We believed our results were less likely to be subject to non-response or attrition bias because our sensitivity analysis yielded similar results when using whole sample and complete cases samples.

We found that individuals with digital device ownership had better cognitive function at baseline and a lower rate of decline than those without internet devices in a four-year follow-up. Our finding is consistent with a study using the English Longitudinal Study of Aging (ELSA) cohort which showed that internet users had better cognitive performance measured by delayed recall compared with internet nonusers [13]. Other results of a protective association between internet use and cognitive function were also reported in other developed countries [12-16]. Although the exact mechanism is not clear, a potential theoretical pathway may be that internet access imparted a reserve against the expression of cognitive impairment [10,26,27]. The cognitively stimulating activities facilitated by digital devices generate a cognitive reserve buffer that promotes resilience so as to prevent or delay the development of cognitive impairment [28]. In addition, we found that the protective effect was larger during the four-year follow-up than that during the two-year follow-up. This is in line with previous studies that show that longer and more intense interventions aimed at the use of digital devices might significantly enhance cognitive functions [29-31].

Although not statistically significant, we observed a trend towards greater longitudinal protective association between cognitive function and both of the digital instruments relative to access to a single device (desktop or cellphone ownership) alone. Different digital devices have different functions and areas of cognition-related application. Computers are mainly used to search for health-related information, such as consulting a doctor at a distance, thus enabling easier access to better and more effective health care for adults [32]. In the context of China, older people tend to use the internet for obtaining health information about proper nutrition, exercise or weight issues and disease management, which contributes to adopting healthier behaviors and making more informed medical decisions. However, cellphones are mainly used to stay in contact with friends, engage in entertainment activities, receive reminders for scheduled visits and for medication instructions, which therefore increase social participation and interaction. Meanwhile, digital device ownership in general stimulates the learning of new things and increases the cognitive demand to master new skills using different digital tools. Future studies focusing on multiple digital devices are warranted to assess the longitudinal protective association between digital devices and cognitive function at mid- and later life.

Stratified analyses indicated that gender and age modified protective association between digital device ownership and cognitive decline, which is consistent with previous studies in other settings [33-36]. Our results suggest that targeting policies on cognitive impairment and dementia prevention around females and earlier age groups might improve their effectiveness. Future studies should focus on understanding mechanisms through which digital device ownership works on slowing cognitive decline.

In summary, our encouraging findings highlighted the importance of promoting the application of internet-based computers, cellphones and other digital devices in middle-aged and elderly Chinese. As more people are connected with multiple digital instruments, the benefit of digital device ownership can be maximized for clinical and health services delivery. A growing group of studies have demonstrated the effectiveness of mobile-based health interventions, such as online cognitive training programs, internet-based conversations and internet or email use [29,30]. A majority of older Chinese have owned a mobile phone at some point, and this high ownership rate suggests that it could become a tool that can be implemented into clinical interventions to reduce cognition loss, especially when connected with the internet. More broadly, our results might promote more widely accessible mobile phones and the continuing penetration of internet access, in order to tackle the increasing challenges of aging in China.

### Limitations

Our study had several limitations. First, the observational nature of our study limited our ability to investigate the causal relationship between digital device ownership and cognitive decline. The results should not be interpreted as the long-term effect of digital device ownership on reducing cognitive decline. Our results also didn’t determine any mechanistic basis behind the observed link between digital device ownership and subsequent cognitive trajectories. Rather, the longitudinal protective association between digital device ownership and cognitive decline found in the present study underscored the need for research to capitalize on new digital technologies to slow cognitive decline.

Second, due to data feasibility, our analysis was limited to digital device ownership without taking into account digital device use. This might limit our ability to understand how digital devices work against cognitive decline. Nevertheless, previous studies have shown that digital device ownership demonstrated a high correlation with use of digital devices [37,38].

Third, because of data availability, the definition of cellphone ownership in our study referred to a mobile phone both with and without internet connection. This might limit our ability to explore the mechanism via which (internet-based or not) the cellphone had a protective association with cognition. Meanwhile, we highlighted that different digital devices have different functions and areas of cognition-related application. Computers are mainly used to search online for health-related information, while cellphones are mainly used to increase social participation and interaction via telephone calls and messages. Interestingly, we found that the magnitude of protective association between computers and cognition was about the same as that between cellphones and cognition.

Fourth, our digital device ownership was only measured at baseline so information on the potential change of digital device ownership was not available. Nevertheless, the fact that our data already had a high baseline ownership rate of single devices (65%) and of both of the devices (16%), that made it less likely that the limitation of only measuring digital access at baseline would lead to differential bias in our results.

### Conclusions

Previous studies using CHARLS cohorts, including one of our own, have demonstrated digital access is associated with better physical health and better outcomes of chronic disease management [39-41]. The national representativeness of CHARLS adds to the robustness of the results, indicating digital devices as a platform for health management. This present study found the longitudinal protective association between digital device ownership and cognitive function. Findings from this study underscored the importance of digital devices as a platform for cognitively stimulating activities to delay cognitive decline. Future studies focusing on use of digital devices are warranted so as to investigate digital devices as a protective factor against cognitive decline at mid- and later life.

## Appendix

Multimedia Appendix 1 Details of interviewer recruitment, training and material preparation, quality control and data cleaning of CHARLS.

Multimedia Appendix 2 Characteristics of the three groups of participants by digital device ownership.

Multimedia Appendix 3 Adjusted association between digital device ownership and cognitive function by gender.

Multimedia Appendix 4 Adjusted association between digital device ownership and cognitive function by age.

Multimedia Appendix 5 Sensitivity analyses restricted analytic cohorts to those participated in all survey waves.

## Abbreviations

CHARLS: China Health and Retirement Longitudinal Study
ELSA: English longitudinal Study of Aging
