# Supplementary material for: Effects of Digital Device Ownership on Cognitive Decline in a Middle-Aged and Elderly Population: Longitudinal Observational Study
Source: J Med Internet Res. 2019 Jul 29;21(7):e14210. doi: 10.2196/14210 (PMC6690159; doi:10.2196/14210)
Supplement: Multimedia Appendix 1 [file jmir_v21i7e14210_app1.pdf]

Multimedia Appendix 1 Implement Details (from CHARLS user guide, available at: http://opendata.pku.edu.cn/dataset.xhtml?persistentId=doi:10.18170/DVN/IZU8OF)

A1. Interviewer recruitment

After two pretests, we decided to recruit the staff independently. We needed one mapper/lister and 2 interviewers for each county so our task was to recruit 150 mappers/listers and 300 interviewers. To automate this process, we designed a web-based recruitment system where job applicants filled in relevant information online and initial screening was conducted. The staff at the Institute of Social Science Surveys of Peking University helped us to advertise the positions at help-wanted websites, both at national websites and provincial sites, and at college Bulletin Board System (BBS). In many cases, we relied on colleagues in local universities to recruit their students as interviewers. Most of the mappers/listers and interviewers were university students in their earlier 20s. The criteria used in selecting mappers/listers and interviewers were their stated willingness to work hard and any previous field experience. Also communication ability was emphasized, particularly knowledge of local dialects that were likely to encounter in the field.

A2. Training and material preparation

Training of mappers/listers and interviewers were each divided into 5 classes and were all conducted in Beijing. The trainings of mappers/listers were conducted in May, each one ran for 5 days. Lectures were given in the mornings and mappers/listers were brought to a nearby community for practice every afternoon. We deliberately choose a community with complicated building structure. Every morning a test was conducted to test their knowledge learned the previous day. The mappers/listers immediately went to the field after training. The trainings of interviewers were conducted in June and July, each one running for 9 days, with DVDs. The first 7 days were lectures and in-class practices. Every day at the end of the class a live interview was conducted with a convenience sample. As in the training of mappers/listers, every morning a test was conducted online on what they learned the previous day. At the 8th day of the training, interviewers were brought to a nearby community and conducted live interviews the way they would in the field. On the last day they went back to the classroom for a summary session. They were first briefed by our quality control team of their performance based on real data that was transmitted from their PC to our server. Then they exchanged their experience from the previous day. At last, they were issued training certificates and had a farewell lunch. Additional trainings were conducted when we needed extra interviewers in cases when interviewers quit or fired. Because we are conducting multiple trainings before the actual field work, we wanted a set of standardized training materials to ensure that all interviewers receive the same training no matter where and when they are trained. The research team and field staff started to produce a set of training manuals since May 2010. They also wrote scripts for various parts of the training course and went through several iterations. These training materials were used in the August pretest in Beijing. The training sessions, conducted by the research team, were recorded and what was spoken was transcribed and compared with the written scripts for further revisions. After the scripts were edited, a set of training videos (DVDs) were made with the help of the Peking

University TV station. In the Langfang pretest in November, we relied mainly on the training videos to test the effectiveness of the DVD-based training. Following this pretest, we adjusted the timing of each DVD, changed the order of the training sessions, and streamlined the procedure. All the training scripts were revised and DVDs were then remade at Peking University's TV station.

A3. Quality control

We made use of the CAPI system to perform quality checks during the field work, using four main methods: GPS matching, data checking, recording and checking interviews and calling back. For GPS matching, we compared the GPS information collected during mapping/listing with that collected at the interviewing stage. We also used photos of the dwelling taken using mapping/listing and at the interview to ensure we located the sampled households. During the survey, a programmer in the CHARLS central headquarters would check the data of all interviewers, looking for excessively missing data and excessively short interviews. In addition the first two households interviewed by each interviewer were be selected for checking the sound recording, to ensure that the interview took place and for certain sections, that questions were being asked correctly and well.. If the sound recording checking did not work because of a technical problem or for other reasons, these households were to be called back by central office staff. For example, data might have indicated that a number of respondents had no job during his or her entire life, but it may have been due to interviewer mistakes. Another example checked was cases in which a respondent missed an entire section of the interview, such as on wealth. The households that failed to do the sound recording for technical or other reasons were contacted, by phone if possible, to see if they in fact had been visited by CHARLS interviewers and the data properly collected. If any misconduct was found during data checking, sound recording checking or calling back checking, we expanded the samples to check of that specific interviewer.

A4. Data cleaning

In addition to coding the occupation and sectors discussed above, we did some basic cleaning of the data and performed further data checks after the field work. We checked skip patterns in the data, some questions should not be asked based on previous answers. For instance, once a respondent tells us that a doctor had diagnosed his or her hypertension, the question of whether the respondent knew that he or she had hypertension should be skipped. We checked all skip patterns in the data and corrected the data which contained incorrect skip patterns (if this existed, it was generally from CAPI programming errors). Following this, we checked for data inconsistencies. A good example of this would be a respondent reporting wage income in the work module, but not reporting income in the individual income module. We will conduct further checks as we obtain feedback from users.
