# Supplementary material for: Effects of Digital Device Ownership on Cognitive Decline in a Middle-Aged and Elderly Population: Longitudinal Observational Study
Source: J Med Internet Res. 2019 Jul 29;21(7):e14210. doi: 10.2196/14210 (PMC6690159; doi:10.2196/14210)
Supplement: Multimedia Appendix 2 [file jmir_v21i7e14210_app2.pdf]

Multimedia Appendix 2. Characteristics of the three groups of participants by digital device ownership

	No Desktop or Cellphone N=2598	Desktop or Cellphone Alone N=8711	Desktop and Cellphone Both N=2148	Joint Test P value
Age, mean (SD)	65.07 (9.27)	57.58 (8.79)	55.25 (8.14)	<0.001
Male sex	1263 (48.6%)	4321 (49.6%)	1006 (46.8%)	0.07
Educational level				<0.001
Illiterate	1113 (42.8%)	2013 (23.1%)	214 (10.0%)	
Part of primary school	550 (21.2%)	1670 (19.2%)	242 (11.3%)	
Primary school	506 (19.5%)	2080 (23.9%)	351 (16.3%)	
Middle school	303 (11.7%)	1989 (22.8%)	632 (29.4%)	
High school or above	126 (4.8%)	958 (11.0%)	709 (33.0%)	
Martial status				<0.001
Married or Partnered	519 (20.0%)	925 (10.6%)	150 (7.0%)	
Otherwise	2079 (80.0%)	7786 (89.4%)	1998 (93.0%)	
Rural residence, mean (SD)	0.68 (0.47)	0.64 (0.48)	0.26 (0.44)	<0.001
Smoke				0.00
Current	812 (31.3%)	2798 (32.1%)	595 (27.7%)	
Former	252 (9.7%)	807 (9.3%)	178 (8.3%)	
Never	1533 (59.0%)	5105 (58.6%)	1375 (64.0%)	
Alcohol drink				0.09
Current	809 (31.1%)	2922 (33.6%)	761 (35.4%)	
Former	251 (9.7%)	747 (8.6%)	142 (6.6%)	
Never	1538 (59.2%)	5038 (57.9%)	1245 (58.0%)	
Ever had high blood pressure, mean (SD)	0.31 (0.46)	0.26 (0.44)	0.27 (0.44)	<0.001
Ever had diabetes, mean (SD)	0.07 (0.25)	0.06 (0.24)	0.08 (0.27)	0.01
Ever had stroke, mean (SD)	0.03 (0.17)	0.03 (0.16)	0.02 (0.14)	0.07
