# Supplementary material for: Effects of Digital Device Ownership on Cognitive Decline in a Middle-Aged and Elderly Population: Longitudinal Observational Study
Source: J Med Internet Res. 2019 Jul 29;21(7):e14210. doi: 10.2196/14210 (PMC6690159; doi:10.2196/14210)
Supplement: Multimedia Appendix 3 [file jmir_v21i7e14210_app3.pdf]

Multimedia Appendix 3. Adjusted association between digital device ownership and cognitive function by gender

Panel A. Independent Effect of Desktop	Coefficients (95% CI)	*P* value	n
**Baseline difference**			
Male	0.11 (0.06, 0.15)	<.001	16730
Female	0.11 (0.06, 0.15)	<.001	17226
**Longitudinal protective association in 2 years**			
Male	0.01 (-0.05, 0.06)	.80	16730
Female	0.03 (-0.02, 0.08)	.20	17226
**Longitudinal protective association in 4 years**			
Male	0.03 (-0.05, 0.06)	.31	16730
Female	0.08 (0.03, 0.13)	.002	17226
Panel B. Independent Effect of Cellphone	Coefficients (95% CI)	*P* value	n
**Baseline difference**			
Male	0.11 (0.06, 0.15)	<.001	16730
Female	0.10 (0.06, 0.15)	<.001	17226
**Longitudinal protective association in 2 years**			
Male	0.02 (-0.03, 0.07)	.50	16730
Female	0.00 (-0.05, 0.05)	.95	17226
**Longitudinal protective association in 4 years**			
Male	0.05 (0.00, 0.10)	.04	16730
Female	0.07 (0.02, 0.12)	.003	17226

Notes: adjusted for demographic (age, education, marriage, rural or urban residence) and health behavior (smoke, drink) as well as health condition risk factors (self-reported hypertension, diabetes, and stroke).
