# Supplementary material for: Effects of Digital Device Ownership on Cognitive Decline in a Middle-Aged and Elderly Population: Longitudinal Observational Study
Source: J Med Internet Res. 2019 Jul 29;21(7):e14210. doi: 10.2196/14210 (PMC6690159; doi:10.2196/14210)
Supplement: Multimedia Appendix 4 [file jmir_v21i7e14210_app4.pdf]

Multimedia Appendix 4. Adjusted association between digital device ownership and cognitive function by age

| ***Panel A. Independent Effect of Desktop*** | Coefficients (95% CI) | *P* value | n |
|---|---|---|---|
| **Baseline difference** | | | |
| Aged 45 and 64 | 0.12 (0.08, 0.15) | <.001 | 25925 |
| Aged 65 and above | 0.17 (0.08,0.25) | <.001 | 8031 |
| **Longitudinal protective association in 2 years** | | | |
| Aged 45 and 64 | 0.02 (-0.02, 0.06) | .31 | 25925 |
| Aged 65 and above | 0.00 (-0.10, 0.09) | .91 | 8031 |
| **Longitudinal protective association in 4 years** | | | |
| Aged 45 and 64 | 0.05 (0.01, 0.08) | .03 | 25925 |
| Aged 65 and above | -0.01 (-0.12, 0.08) | .77 | 8031 |
| ***Panel B. Independent Effect of Cellphone*** | Coefficients (95% CI) | *P* value | n |
| **Baseline difference** | | | |
| Aged 45 and 64 | 0.11 (0.07, 0.15) | <.001 | 25925 |
| Aged 65 and above | 0.10 (0.06, 0.16) | <.001 | 8031 |
| **Longitudinal protective association in 2 years** | | | |
| Aged 45 and 64 | -0.02 (-0.06, 0.03) | .42 | 25925 |
| Aged 65 and above | 0.01 (-0.04, 0.07) | .62 | 8031 |
| **Longitudinal protective association in 4 years** | | | |
| Aged 45 and 64 | 0.02 (-0.03, 0.06) | .43 | 25925 |
| Aged 65 and above | 0.02 (-0.04, 0.08) | .60 | 8031 |

Notes: adjusted for demographic (sex, education, marriage, rural or urban residence) and health behavior (smoke, drink) as well as health condition risk factors (self-reported hypertension, diabetes, and stroke).
