# Supplementary material for: Effects of Digital Device Ownership on Cognitive Decline in a Middle-Aged and Elderly Population: Longitudinal Observational Study
Source: J Med Internet Res. 2019 Jul 29;21(7):e14210. doi: 10.2196/14210 (PMC6690159; doi:10.2196/14210)
Supplement: Multimedia Appendix 5 [file jmir_v21i7e14210_app5.pdf]

Multimedia Appendix 5. Sensitivity analyses restricted analytic cohorts to those participated in all survey waves

Panel A. Desktop Ownership	Without Desktop	With Desktop	P
Baseline difference	reference	0.11	<.001
(95% CI)		(0.07, 0.15)	
Changes in 2 years	-0.03	-0.01	.50
(95% CI)	(-0.04, -0.01)	(-0.05, 0.02)	
Changes in 4 years	-0.16	-0.10	.002
(95% CI)	(-0.18, -0.15)	(-0.14, -0.06)	
Number of Observations	29727		
Panel B. Cellphone Ownership	Without Cellphone	With Cellphone	P
Baseline difference	reference	0.10	<.001
(95% CI)		(0.07, 0.13)	
Changes in 2 years	-0.03	-0.02	.82
(95% CI)	(-0.06, 0.00)	(-0.04, -0.01)	
Changes in 4 years	-0.20	-0.14	<.001
(95% CI)	(-0.24, -0.17)	(-0.16, -0.12)	
Number of Observations	29727		

Notes: adjusted for demographic (age, sex, education, marriage, rural or urban residence) and health behavior (smoke, drink) as well as health condition risk factors (self-reported hypertension, diabetes, and stroke).
